# Osteogenic Surface Modification Based on Functionalized Poly-*P*-Xylylene Coating

**DOI:** 10.1371/journal.pone.0137017

**Published:** 2015-09-17

**Authors:** Chih-Hao Chang, Shu-Yun Yeh, Bing-Heng Lee, Chia-Jie Chen, Chiao-Tzu Su, Yen-Ting Lin, Chien-Lin Liu, Hsien-Yeh Chen

**Affiliations:** 1 Department of Orthopedics, National Taiwan University Hospital and National Taiwan University College of Medicine, Taipei, Taiwan; 2 Department of Chemical Engineering, National Taiwan University, Taipei, Taiwan; 3 Department of Orthopaedics and Traumatology, Taipei Veterans General Hospital, Taipei, Taiwan and School of Medicine, National Yang-Ming University, Taipei, Taiwan; Harvard Medical School, UNITED STATES

## Abstract

The biotechnology to immobilize biomolecules on material surfaces has been developed vigorously due to its high potentials in medical applications. In this study, a simple and effective method was designed to immobilize biomolecules via amine-N-hydroxysuccinimide (NHS) ester conjugation reaction using functionalized poly-*p*-xylylene coating on material surfaces. The NHS ester functionalized coating is synthesized via chemical vapor deposition, a facile and solvent-less method, creating a surface which is ready to perform a one-step conjugation reaction. Bone morphogenetic protein 2 (BMP-2) is immobilized onto material surfaces by this coating method, forming an osteogenic environment. The immobilization process is controlled at a low temperature which does not damage proteins. This modified surface induces differentiation of preosteoblast into osteoblast, manifested by alkaline phosphatase (ALP) activity assay, Alizarin Red S (ARS) staining and the expression of osteogenic gene markers, *Alpl* and *Bglap3*. With this coating technology, immobilization of growth factors onto material surface can be achieved more simply and more effectively.

## Introduction

As the technology of material science progresses, not only the design of bulk materials but also the technologies of surface modification have become more and more valued. The functionalities of biomaterials with surface modification have made bioengineered implants more promising. Specific functions of biomaterials were achieved by surface modifications using different biomolecules onto material surfaces. For instance, controlling fundamental cellular process on material surfaces is one of the most essential functions for bioengineered implants. Growth factors are key factors in many fundamental cellular processes such as cell proliferation or differentiation. As a result, growth factors are used widely for surface modifications. Yang et al. applied physically-adsorbed fibronectin to surfaces of poly lactic acid (PLA) films and poly lactic-co-glycolic acid (PLGA) scaffold and demonstrated that cells showed well adhesion and spreading on the fibronectin-adsorbed materials [1]. Karageorgiou et al. reported the covalently-bonded bone morphogenetic protein-2 (BMP-2) on the surface of silk fibroin films successfully induced osteogenic differentiation of human bone marrow stromal cells [2]. Among those surface modification methods, immobilization of biomolecules has become one of the most emerging fields of study due to the promising biological benefits for modified biomaterials. For instance, immobilized versions of proteins and growth factors have been shown to be superior for prolonged availability to induce cellular outcomes (thus restricting delivery to the local implant region) compared to the delivery of a direct injection or weakly bound proteins, which are rapidly degraded through endocytosis pathways; in addition, the delivery of large quantities increases cost and can damage cells and tissues [3–5]. Various immobilization approaches are not applicable on different types of materials [5–8] and usually require complex procedures to perform surface modifications [9, 10].

In this study, we introduce a facile and versatile approach to immobilize growth factor protein of BMP-2 by using N-hydroxysuccinimide (NHS) ester-functionalized poly-*p*-xylylene coating, which is prepared via chemical vapor deposition (CVD) polymerization. Poly-*p*-xylylene has been known as a coating material with robust adhesion and high biocompatibility, and has been used for coating implantable medical devices [11–14]. Later, variable functionalized poly-*p*-xylylene coatings were developed and used for immobilizing molecules onto material surfaces [15]. Using CVD polymerization, functionalized poly-*p*-xylylene coatings can be applied to various substrate materials [16]. NHS ester-amine coupling reaction is one of the reactions used most widely to bind biomolecules, especially proteins, and thus becoming an important binding mechanism for immobilization of biomolecules onto material surfaces [17–19]. The resulting coating provides a one-step approach to install NHS ester anchoring sites to material surfaces, and is equally applicable to various materials, including metals, polymers, and silicon, similar to others from the poly-*p*-xylylene family [20, 21], and shows excellent stability and adhesion properties [22]. In addition, the modified material surfaces are readily available to perform orthogonal conjugations of NHS ester-amine coupling reaction at a temperature which does not damage proteins. This technique has provided the most straightforward route relying on the formation of an amide bond that is accessible for conjugation without denaturing the protein [23–25]. The conjugation between this NHS ester-functionalized coating and BMP-2 was confirmed by surface characterization with X-ray photoelectron spectroscopy (XPS) and infrared reflection absorption spectroscopy (IRRAS), and the quantitation of protein immobilization was measured by quartz crystal microbalancing (QCM) analysis. The osteogenesis-inducing ability of the BMP-2 modified surface was proved by alkaline phosphatase (ALP) activity assay, Alizarin Red S (ARS) staining and the expression of osteogenic gene markers, *Alpl* (ALP) and *Bglap3* (osteocalcin), were also measured to verify the cell differentiation from preosteoblast to osteoblast.

## Materials and Methods

### Materials

The following materials were obtained commercially and used as received unless otherwise noted: [2,2]paracyclophane (Jiangsu Miaoqiao Synthesis Chemical Co., China, 98%), aluminum chloride (Alfa Aesar, 99%), dichloromethane (Macron Chemicals, USA), anhydrous magnesium sulfate (J.T. Baker, USA, 99.5%), trifluoroacetic anhydride (Sigma-Aldrich, 99%), Hydrochloric acid (Sigma-Aldrich, 37%), sodium hydroxide (Sigma-Aldrich, 99%), potassium hydroxide (Showa Kako Corp., 85.5%), Tetrahydrofuran (Sigma-Aldrich, 99.9%), N,N’-dicyclohexylcarbodiimide (Sigma-Aldrich, 99%), N-Hydroxysuccinimide (Alfa Aesar, 98%), recombinant human bone morphogenetic protein 2 (355-BM-050/CF, R&D systems, USA), and silicon wafers (Goldeninent Inc., Taiwan). Gold substrates were fabricated on a 4-inch silicon wafer by depositing a 300-Å layer of titanium followed by a 700-Å layer of gold with a thermal evaporator (Kao Duen Technology Co., Taiwan). All silicon substrates were cleaned using a piranha solution (3:1 v/v H_2_SO_4_:H_2_O_2_) before use.

### Synthesis of 4-N-hydroxysuccinimide (NHS) ester-[2.2]paracyclophane

**Fig 1:**
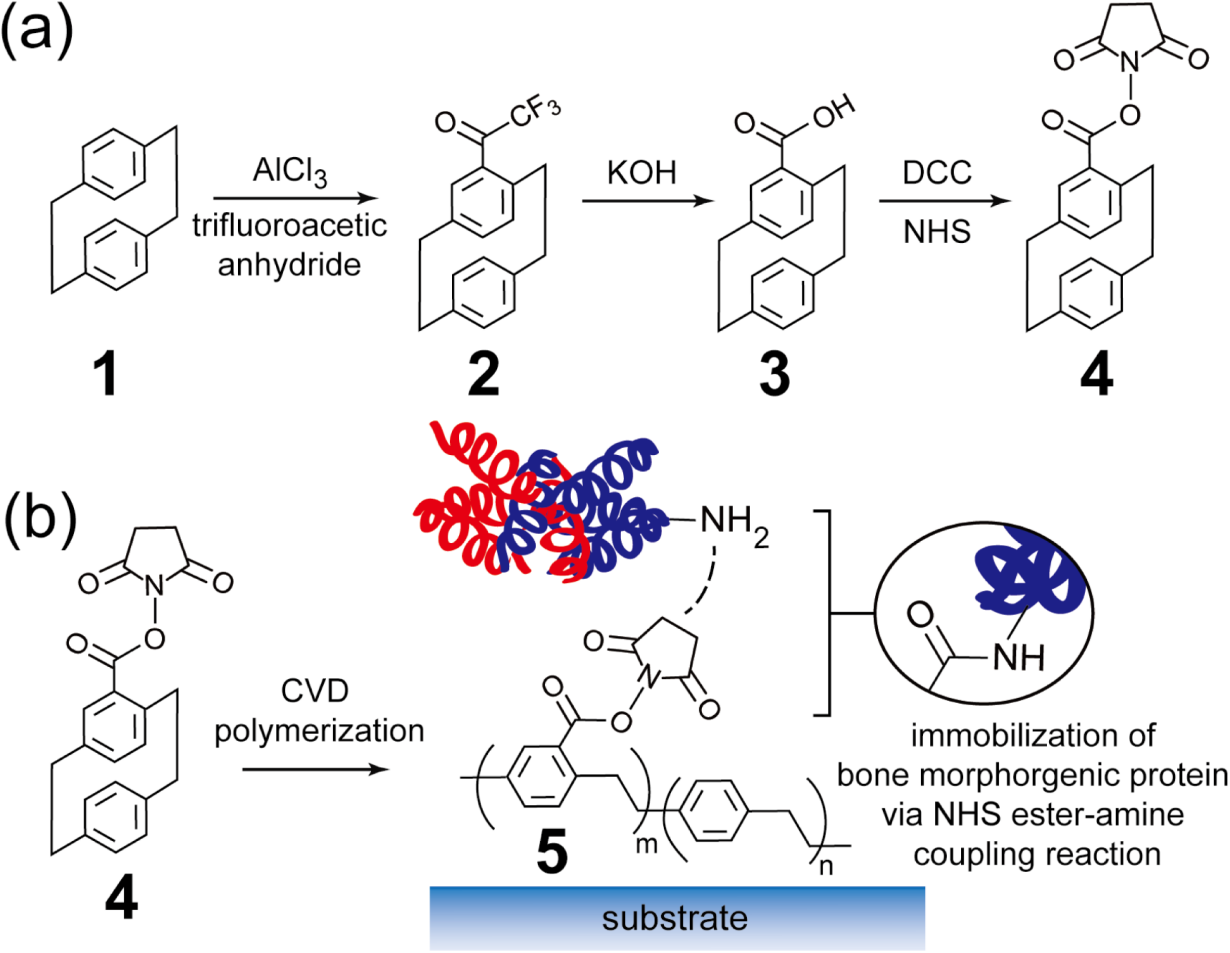
Schematic figure of (a) synthesis of 4-N-hydroxysuccinimide (NHS) ester-[2.2]paracyclophane and (b) immobilization of BMP-2. (a) Synthetic route of 4-N-hydroxysuccinimide ester-[2.2]paracyclophane **4**. (b) CVD polymerization of **4** to poly(4-N-hydroxy-succinimide ester-*p*-xylylene-*co*-*p*-xylylene) (coating **5**) and immobilization of protein by forming an amide bond between protein and coating **5**.

The schematic figure of the reactions was shown in Fig 1(A). 4-N-hydroxysuccinimide ester-[2.2]paracyclophane **4** was synthesized via a three-step procedure. Commercially available [2,2]paracyclophane **1** was first used to produce 4-trifluoroacetyl- [2,2]paracyclophane **2**. Before the reaction, [2,2]paracyclophane **1** and aluminum chloride (AlCl_3_) were dissolved in dichloromethane separately and trifluoroacetic anhydride was added in the aluminum chloride solution and after 15-mins stirring, [2,2]paracyclophane **1** solution was gently added in. The reaction was kept at 0°C for 90 mins. 4-trifluoroacetyl- [2,2]paracyclophane **2** was obtained by being subjected to Friedel-Crafts acrylation in the presence of trifluoroacetic anhydride and aluminum chloride (AlCl_3_). The organic layer was wash with 3 M HCl (2 × 300 mL) and then with deionized water (2 × 300 mL), and dried over MgSO_4_. The yield of this step is 95%. 4-trifluoroacetyl- [2,2]paracyclophane **2** was subsequently hydrolyzed with 10% potassium hydroxide (KOH) solution to produce 4-carboxyl-[2,2]paracyclophane **3**. At the end of this step, HCl was gently added in and the 4-carboxyl-[2,2]paracyclophane **3** was precipitated. (85% yields). Compound **3** and N,N’-dicyclohexylcarbodiimide (DCC) were then dissolved in tetrahydrofuran (THF) and stirred for 20 minutes, followed by adding N-hydroxysuccinimide (NHS) to react for 16 hours. The resulting product was 4-N-hydroxysuccinimide ester-[2.2]paracyclophane **4.**

### Chemical vapor deposition (CVD) polymerization

The synthesis of 4-N-hydroxysuccinimide ester-[2.2]paracyclophane was included in Surpporting Information. Coating **5** was prepared from 4-N-hydroxysuccinimide ester-[2.2]paracyclophane **4** based on a custom-built system, which consists of a deposition chamber, a pyrolysis zone, and a sublimation zone. The starting material, compound **4** was first sublimed at the temperature of 100–120°C and then transported into the pyrolysis zone in which the temperature was raised to 700°C by the introduction of argon carrier gas at a flow rate of 30 sccm. The subsequently formed radicals were transferred into the deposition chamber and the polymerization process occurred on a rotating holder was maintained at 15°C to ensure uniform coating. Throughout the CVD process, a deposition rate at approximate 0.5 Å s^-1^ was monitored on the basis of in situ quartz crystal microbalancing analysis (STM-100/MF, Sycon Instruments, USA) and a reduced pressure of 75 mTorr was regulated to optimize the polymerization.

### Immobilization of bone morphorgenic protein 2 (BMP-2)

To immobilize BMP-2 on the coating **5** modified surface, commercially obtained BMP-2 (355-BM-050/CF, molecular weight: 26 kDa, R&D systems, USA) was dissolved in deionized water. The concentration of BMP-2 solution used throughout this study was 50 μg ml^-1^. The BMP-2 solution was dropped on the coating **5** modified surface directly after CVD polymerization, and kept at 4°C overnight. At the end of the reaction, the treated surfaces were washed by PBS solution at least three times.

### Surface characterization

Infrared reflection absorption spectroscopy (IRRAS) was performed using a Perkin Elmer Spectrum 100 FT-IR spectrometer equipped with a liquid nitrogen cooled MCT detector, and the spectra were corrected for any residual baseline drift. X-ray photoelectron spectroscopy (XPS) was characterized with a Theta Probe X-ray photoelectron spectrometer (Thermal Scientific, UK) using a monochromatized AlKα as the X-ray source at an X-ray power of 150 kW. For obtaining survey spectra, the pass energy of 200.0 eV was used. The XPS atomic analysis was reported based on the atomic concentrations (%) and was compared to the theoretical values calculated on the basis of the structure.

To analyze the amount of protein immobilization on coating **5** quantitatively, a QCM instrument (ANT Technologies, Taiwan) equipped with a flow injection analysis (FIA) device and a continuous frequency variation recording device was employed. The flow rate was controlled by a peristaltic pump connected to the FIA device. An AT-cut piezoelectric quartz disc with a 9 MHz resonant frequency and a 0.1 cm^2^ total sensing area was used as the sensing element of this instrument. BMP-2 or BMP-2 primary (MAB3551, R&D Systems) antibody solution was injected to the system with modified surfaces to analyze the quantity of BMP-2 or BMP-2 primary antibody which immobilized or adsorbed on the surfaces. For the antibody binding experiments, the pumping process was temporarily stopped for 25 min (10 min after injection) to allow binding of BMP-2 and/or antibodies. The QCM analysis was performed at 25°C.

### Cell culture

Murine preosteoblasts (MC3T3-E1 subclone 4, CRL-2593, ATCC, USA) passage 20 were plated at 4 · 10^4^ cells cm^-2^ in 96-well cell culture plates using basal proliferation medium comprised of Minimum Essential Medium α (MEM α, nucleosides, no ascorbic acid, Life Technologies, USA) supplemented with 10% Fetal Bovine Serum (FBS, heat inactivated, qualified, US origin, Life Technologies, USA) for 24 hours. Starting the next day, cells were incubated in standard osteogenic medium comprised of MEM α supplemented with 10% FBS, 50 μg ml^-1^ ascorbic acid (Sigma-Aldrich, USA), 10 nM dexamethasone (Sigma-Aldrich, USA), and 10 mM β-glycerophosphate (Sigma-Aldrich, USA). Osteogenic media were changed twice per week. As for all experiments, MC3T3-E1 cells were examined and quantified at 4, 7, 11, 14 and 21 d for ALP activity and matrix mineralization, as described in details below. For all the examinations outlined below, two independent experiments were conducted with at least three repeats in each experiment.

### ALP activity assay and ARS staining

ALP activity of MC3T3-E1 cells were examined on days 4, 7 and 11. For ALP activity assay, MC3T3-E1 cells were fixed in 4% paraformaldehyde (Sigma-Aldrich, USA) for 30 min, and then rinsed with deionized water three times. Fixed cells were treated with nitro blue tetrazolium/5-bromo-4-chloro-3-indolyl phosphate (NBT/BCIP solution, Sigma-Aldrich, USA) for 30 min. ALP positive cells were visualized as deep blue under the optical microscope. For quantification of ALP activity, cells were washed once with PBS solution and 70μl Alkaline Phosphatase Yellow (pNPP) Liquid Substrate System for ELISA (Sigma-Aldrich, USA) was added. The cells were then incubated for 5 min in the dark. Finally, ALP activity was analyzed by an ELISA reader at 405nm wavelength.

Calcium deposits produced by tested MC3T3-E1 cells were examined on days 7, 14 and 21 by ARS staining assay. For Alizarin Red S staining, MC3T3-E1 cells were washed with PBS solution and fixed in 4% paraformaldehyde for 30 min. Cells were then stained with 1% Alizarin Red S (ARS, Sigma-Aldrich, USA) pH 4.1 for 30 min followed by washing with excess deionized water. For the quantification of ARS staining, stained cells were destained for 15 min with 10% cetylpyridinium chloride (Sigma-Aldrich, USA) and measured at 562 nm wavelength using a microplate reader.

### Gene expression assay

For analyzing the gene expression of MC3T3-E1 cells cultured on different surface, the total RNA was prepared using the Direct-zol RNA Miniprep Kit (Zymo Research, USA) including DNase digestion. RNA concentration was determined through photometric measurement on the Nanodrop 1000 Spectrophotometer (Thermo, USA) and validated RNA quality through formaldehyde-agarose gel electrophoresis [26]. Equal amounts of RNA (200 ng per sample) were reverse transcribed using TaqMan Reverse Transcription Reagents (N8080234, Life Technologies, USA) for first-strand cDNA synthesis. The qRT-PCR were performed with the first-strand cDNA corresponding to 20 ng of total RNA and the Taqman Universal PCR Master Mix (Life Technologies, USA) as well as one of the following Taqman predeveloped assay reagents for mouse:

1. *Alpl* (Mm00475834_m1, FAM/MGB probe, Life Technologies, USA)
2. *Bglap3* (Mm00647982_gh, FAM/MGB probe, Life Technologies, USA)
3. *Gapdh* (Mm99999915_g1, FAM/MGB probe, Life Technologies, USA)

Gene transcription levels of *Alpl*, *OCN* and *Gapdh* were analyzed by real-time PCR (Taqman gene expression assays) on ABI PRISM 7900HT Sequence Detection System (Life Technologies, USA). The housekeeping gene, *Gapdh*, was used as an endogenous control. Each PCR reaction was run in triplicate, and no-template controls were included for each primer pair. For data analysis of mRNA expression, the results from day 4, 7, 11, 14, and 21 were further divided by the basal expression level on day 0. The fold-changes in the abundance of the *Alpl* and *Bglap3* transcripts between various samples were determined using the ΔΔCt method [27].

### Statistical Analysis

Statistical analyses were performed with SPSS 20 (IBM, Chicago, IL, USA). One-way ANOVA combined with a parametric paired *t* test was utilized to determine the significance of ALP activity, matrix mineralization and gene expression. The level of significance was set at *P < 0.05, and high significance was set at **P < 0.01.

## Results and Discussion

### Surface modification method

The immobilization of growth factors can provide an improved interaction and stimulated cellular activity between the host tissue and the biomaterials after implanting [3]. The physical adsorption is the simple way for growth factors coating on the surface of the biomaterial. However, the approach may not be sufficient to promote long-term implantation. Since the molecules are held by weak interactions by the process, many of the molecules will diffuse from the surface without eliciting the desired response [3, 28–30]. Another approach is to design an covalently immobilization scheme. The covalently immobilization technique is to chemically attach the biomolecules to the surface of the implant. This way can promote a specific and controllable interaction between the implant and the host tissue. The immobilization of a particular type of protein or biomolecules would be more likely to produce specific receptor-ligand interactions that could be used to produce a desired outcome [31–36]. Here we report the immobilization of rhBMP-2 on amine-NHS ester functionalized poly-p-xylylene coating to assure marked osteogenic activity, which will be applied for bone-regeneration purposes.

In the experiment, the starting material was first prepared following the synthesis route shown in Fig 1(A). The resulting 4-N-hydroxysuccinimide ester-[2.2]paracyclophane (compound **4**) present in the supernatant liquid was further purified through a column chromatography process by introducing an eluent containing EA and hexane with the volume ratio of 1 to 2, and the overall yield was found to be 65%. The product was analyzed by nuclear magnetic resonance (NMR) spectroscopy. ^1^H NMR (500 MHz, CDCl_3_, TMS): *δ* = 2.86–2.92 (5H; CH_2_), 3.00–3.08 (2H;CH_2_), 3.13–3.18 (4H;CH_2_), 3.97–4.02 (1H;CH_2_), 6.47–6.49 (1H;CH), 6.53–6.54 (2H;CH), 6.60–6.62 (1H;CH), 6.72–6.76 (2H;CH), 7.31 (1H;CH). The synthesis of poly(4-N-hydroxy-succinimide ester-*p*-xylylene-*co*-*p*-xylylene) **5** (here after referred as coating **5**) was performed by using a self-designed CVD system that constituted of a sublimation zone, a pyrolysis zone, and a deposition chamber. Compound **4** was first sublimated at a temperature of 100–120°C and was then transferred to the pyrolysis zone by a stream of argon carrier gas at a flow rate of 30 sccm. The temperature in pyrolysis zone was adjusted to 600°C. After pyrolysis, the radicals were then transferred into the deposition chamber and polymerized onto materials of interest, where a uniform deposition of coating **5** was formed on material surfaces. A pressure of 75 mTorr was preserved throughout the CVD polymerization process, and the deposition rates were regulated at approximately 0.5–1 Å s^-1^. The substrate materials used throughout this study were gold for surface analysis and cell culture plates for culturing MC3T3-E1 preosteoblasts.

With the NHS ester functionalized surface, proteins or other amine-derived molecules can be anchored onto material surfaces easily under a mild reaction condition without any solvent or high temperature that may make proteins denatured. The reaction temperature, which was controlled at 4°C, protected the quality of proteins immobilized on material surface, and thus maintained the function of proteins after the immobilization process.

### Surface characterization

**Fig 2:**
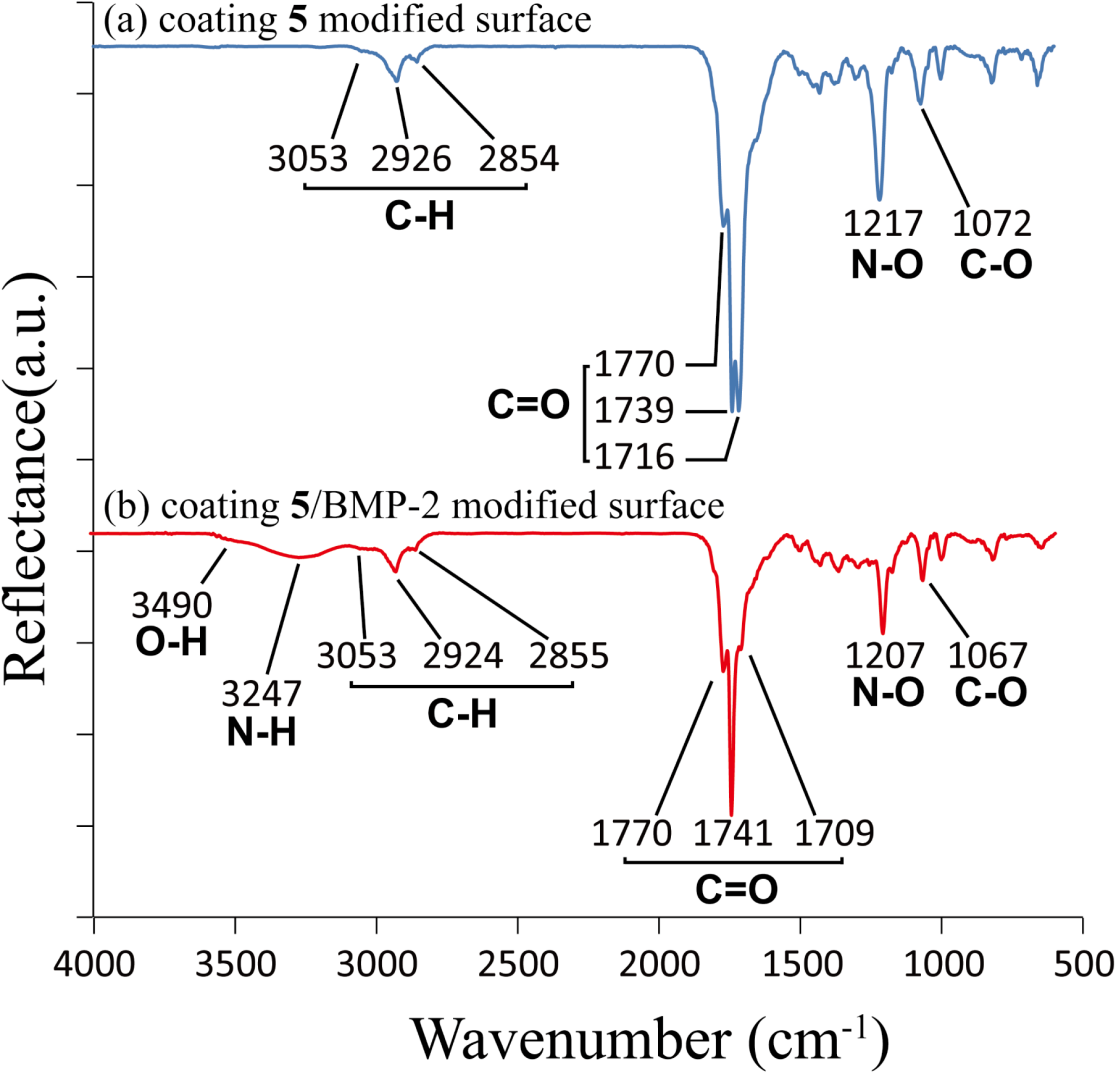
IRRAS characterization of (a) coating 5 modified surface and (b) coating 5/BMP-2 modified surface. Both of the modifications were on a gold-coated silicon substrate. Three significant peaks, which are characters of asymmetric stretching bands of NHS ester C = O were detected as 1770, 1739, and 1716 cm^-1^ in S1(a). Peaks at 1217 cm^-1^ and 1072 cm^-1^ were attributed to N-O and C-O stretch, respectively. In S1 (b), peak of N-O (1207 cm^-1^) and C-O (1067 cm^-1^) stretch were reduced and one of the C = O peak (1716 cm^-1^ in (a) and 1709 cm^-1^ in (b)) strongly reduced after immobilization of BMP-2. The characterization peaks of BMP-2 appeared on the bands around 3247 cm^-1^ for N-H and 3490 cm^-1^ for O-H.

**Fig 3:**
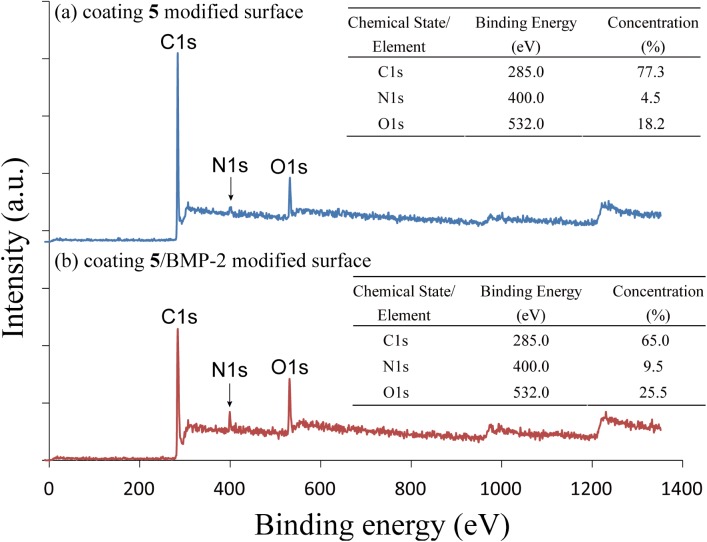
XPS survey spectra of (a) coating 5 modified surface and (b) coating 5/BMP-2 modified surface. The atomic concentration of C, N and O are 77.3%, 4.55% and 18.22% on the coating **5** modified surface and 65.0%, 9.5% and 25.5% on the coating **5**/BMP-2 modified surface. The N composition significantly increased due to immobilization of BMP-2.

Characterizations using a combination of IRRAS and XPS have confirmed the characteristic band vibrations and chemical compositions of coating **5**. As shown in Fig 2(A), characteristic peak at 1716 cm^-1^ 1739 cm^-1^ and 1770 cm^-1^ were the carbonyl stretches from NHS ester groups. The IRRAS results after immobilization of BMP-2 are shown in Fig 2(B). The ratio between 1709 cm^-1^ and 1741 cm^-1^ carbonyl stretches declined and N-H and O-H characteristic band around 3247 cm^-1^ and 3490 cm^-1^ were detected due to the proteins immobilized on surface. The XPS data further confirmed the immobilization of BMP-2. As shown in Fig 3, the atomic concentration of the coating 5 modified surface is 77.3 atom% for carbon, 4.5 atom% for nitrogen and 18.2 atom% for oxygen. After immobilization of BMP-2 the atomic concentration became 65.0 atom% for carbon, 9.5 atom% for nitrogen and 25.5 atom% for oxygen. The concentration of nitrogen and oxygen both increase because of the immobilization of BMP-2 on the surface.

**Fig 4:**
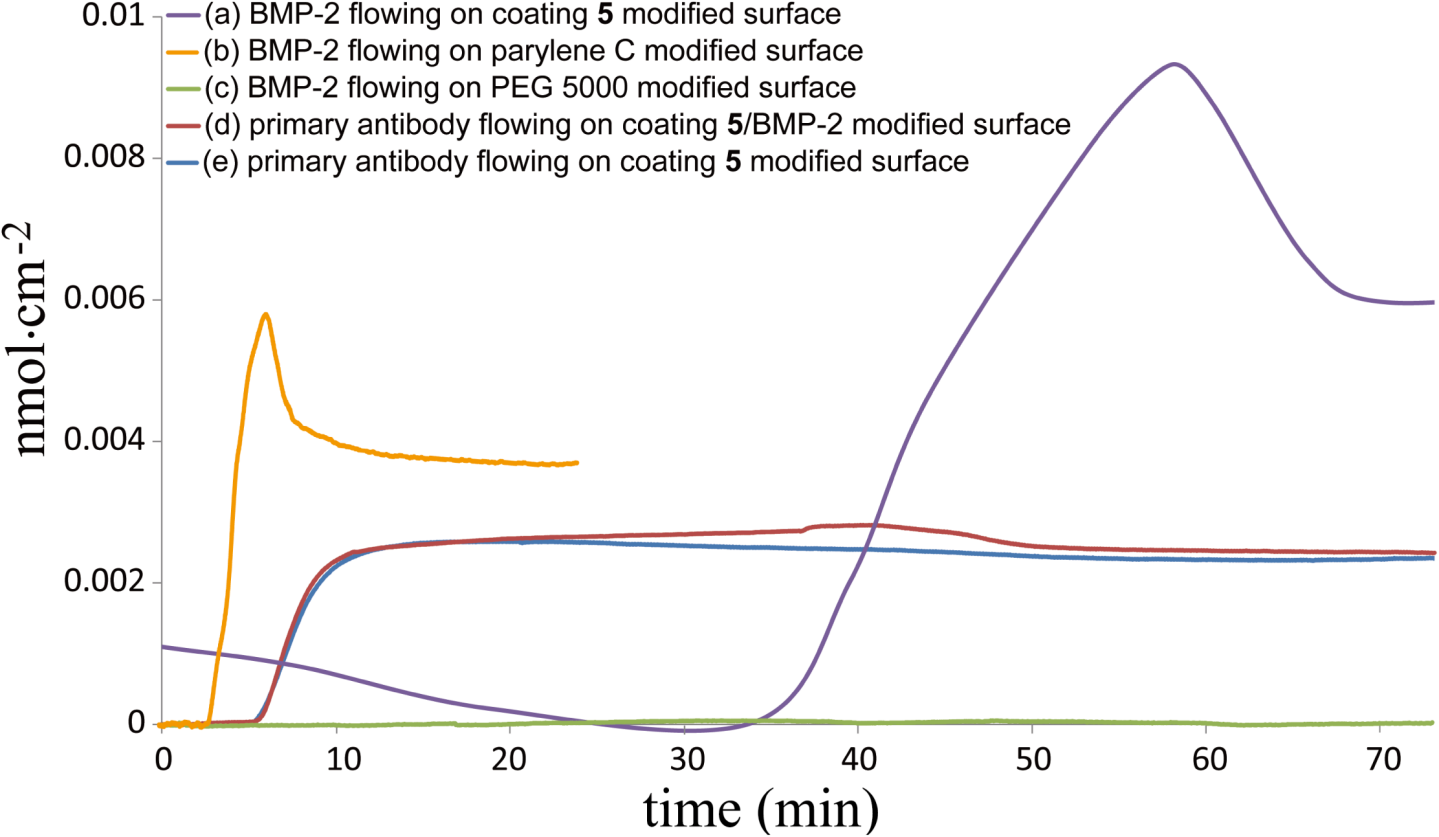
QCM analysis of coating 5 modified surface. Curve (a) is the result of BMP-2 solution injected and flowing through coating **5** modified surface, and at the end of the measurement, the amount of BMP-2 adsorption on coating **5** modified surface is 6.04·10^−12^ mol cm^-2^. Curve (b) is the result of BMP-2 solution injected and flowing through parylene C-modified surface, and at the end of the measurement, the amount of BMP-2 adsorption on parylene C surface is 3.81·10^−12^ mol cm^-2^. Curve (c) is the result of BMP-2 solution injected and flowing through PEG5000 modified surface, and at the end of the measurement, only 1.62·10^−14^ mol cm^-2^ of BMP-2 adsorbed on the surface. Curve (d) is the result of BMP-2 primary antibody solution injected and flowing through coating **5**/BMP-2 modified surface, and at the end of the measurement, the amount of BMP-2 primary antibody adsorption on coating **5** modified surface is 2.43·10^−12^ mol cm^-2^. Curve (e) is the result of BMP-2 primary antibody solution injected and flowing through coating **5** modified surface, and at the end of the measurement, the amount of BMP-2 primary antibody adsorption on coating **5** modified surface is 2.35·10^−12^ mol cm^-2^.

The resulting conjugation efficacy of coating **5** toward BMP-2 and the binding affinity of thereafter immobilized BMP-2 toward human BMP-2 antibody (primary antibody) were further verified by quartz crystal microbalancing (QCM) analysis. The QCM analysis results are demonstrated in Fig 4. BMP-2 or BMP-2 primary antibody solution was injected to the system and flew through the testing surface, then adsorbed or immobilized onto the surface, which caused the curves shifted. The results indicated that a surface concentration of (6.04±0.16)·10^−12^ mol cm^-2^ of BMP-2 was found on coating **5** (Fig 4 curve (a)). On the other hand, low amounts of BMP-2 were found on these controlled surfaces. As shown in curve (b) and (c), the physically adsorbed non-reactive poly(chloro-*p*-xylylene) (parylene C) modified surfaces and the physically repelled poly ethylene glycol (PEG)-modified antifouling surfaces, which are (3.81±0.01)·10^−12^ mol cm^-2^ and (1.62±0.17)·10^−14^ mol cm^-2^, respectively. The binding affinity with respected to the primary antibody was confirmed for the coating **5**/BMP-2 modified surfaces (curve (d)), and compared also to the coating **5** modified surfaces (curve (e)), which are 2.43·10^−12^ mol cm^-2^ and 2.35·10^−12^ mol cm^-2^. To compare curve (a) with (b), the concentration of BMP-2 immobilized on coating **5** modified surface was about 159% of those adsorbed on parylene C surface. According to curve (a) and (e), the number of BMP-2 on coating **5** modified surface was about 3 times to the number of BMP-2 primary antibody on coating **5** modified surface. That is because the molecular size of BMP-2 primary antibody is relatively large to BMP-2, the steric effects are a dominant factor which affected the proteins immobilized on the surfaces. Several studies have demonstrated that crowding effects alter both the affinity and the kinetics of binding of proteins to the surface [37, 38]. The factors of an order of magnitude affect the density of macromolecules presenting on the surface. The results also suggested the binding affinity of BMP-2 toward primary antibody was not influenced by the immobilization process.

### ALP activity assay and ARS staining

**Fig 5:**
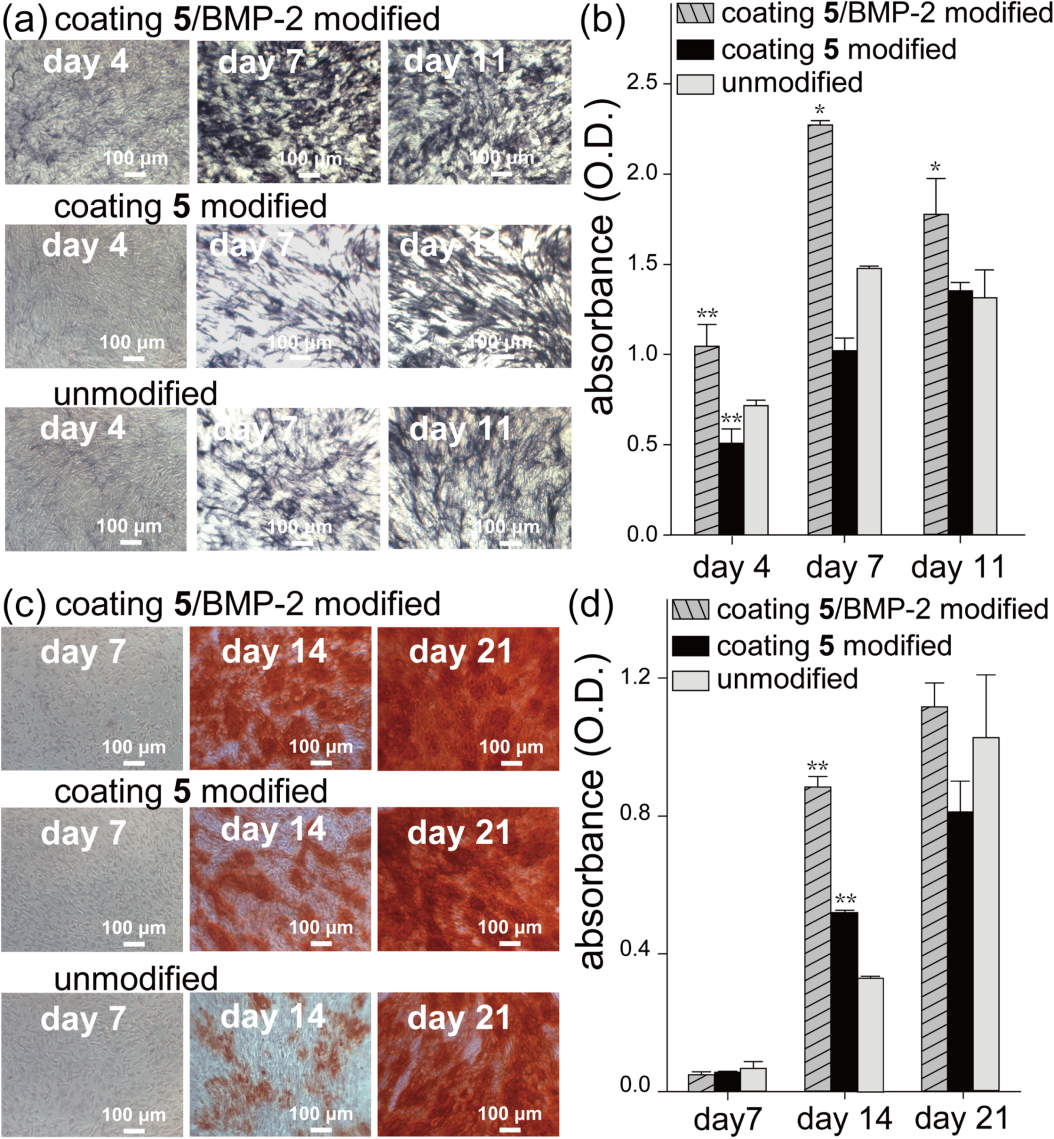
ALP activity assay and ARS staining. ALP activity and calcium deposits of MC3T3-E1 preosteoblasts were evaluated on coating **5**/BMP-2 modified, coating **5** modified and unmodified surface. (a) ALP staining assay and (b) quantification results of ALP at day 4, 7, 11. (c) ARS staining and (d) quantification of calcium deposits on days 7, 14 and 21. The asterisks indicate significant differences (*P<0.05 and **P<0.01) between coating **5**/BMP-2 modified, coating **5** modified and unmodified surface at each time point.

The important biological assessment of the BMP-2 modified surface was finally performed by culturing MC3T3-E1 preosteoblasts on such surfaces for 3 weeks. Separate experiments were conducted either on modified or unmodified surfaces in osteogenic medium. Alkaline phosphatase (ALP) activity and concentration of calcium deposits were monitored. They are the markers for osteogenesis process. The NBT-BCIP solution, a chromogenic phosphatase substrate, which produces blue-colored precipitates, was employed to the ALP staining assay on days 4, 7 and 11. A quantitative analysis was also verified by Alkaline Phosphatase Yellow (pNPP) Liquid Substrate System for ELISA, a reagent develops a soluble yellow reaction product, which can be read at 405 nm. As shown in Fig 5(A), ALP was stained in deep blue and the coating **5**/BMP-2 modified group was significantly darker than other groups from day 4 to day 11. Fig 5(B) displays the quantitative results of ALP staining assay. On day 4, all groups presented low ALP activities relative to other days, and the coating **5**/BMP-2 modified group had the highest ALP activity, signaling the early stage of osteogenesis. From day 7 to day 11, ALP activities in all groups increased and the coating **5**/BMP-2 modified group still expressed the highest ALP activity, indicating that the function of the modified layer acted sustainably. Alizarin Red S (ARS), which forms complex with calcium, was utilized to monitor the calcium deposits on days 7, 14 and 21. ARS staining results are shown in Fig 5(C) and quantification results are shown in Fig 5(D). Calcium deposits, which are important indications of the middle stage of osteogenesis, were not detected by ARS staining on day 7 in any of the groups but appeared in all groups from day 14. The concentration of calcium deposits continued to increase till the end of the experiment period, indicating that the osteogenesis process was coming into the middle stage. According to the results, the coating **5**/BMP-2 modified group displayed the highest amount of calcium deposits among all groups, which represents a higher degree of osteogenesis.

### Gene expression assay

**Fig 6:**
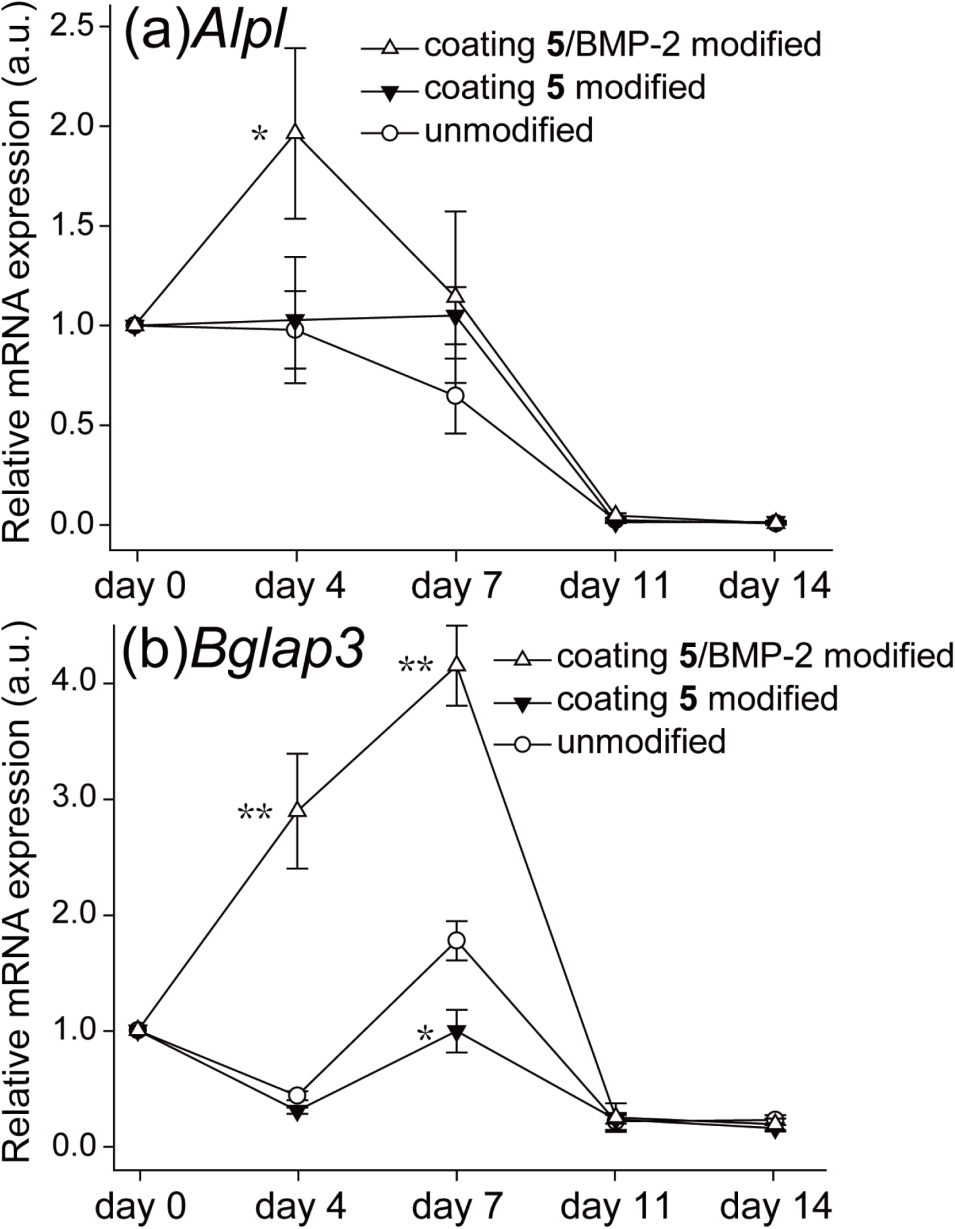
Relative gene expression of (a) Alpl (gene marker of ALP) and (b) Bglap3 (gene marker of osteocalcin). The effect of coating **5**/BMP-2 modified, coating **5** modified and unmodified surface on relative mRNA expression was assayed on days 0, 4, 7, 11, and 14. The asterisks indicate significant differences (*P<0.05 and **P<0.01) between coating **5**/BMP-2 modified, coating **5** modified and unmodified surface at each time point.

To measure the expression level of mRNAs for osteoblast differentiation marker, ALP and osteocalcin, the expression of *Alpl* and *Bglap3* in the MC3T3-E1 preosteoblasts were quantified on days 0, 4, 7, 11 and 14 [39]. For analyzing the gene expression, total RNA of the MC3T3-E1 preosteoblasts was collected and the RNA concentration was determined through photometric measurement. Gene transcription levels of *Alpl* (ALP), *Bglap3* (osteocalcin) and *Gapdh* (housekeeping gene) were analyzed by real-time quantitative reverse transcription polymerase chain reaction (qRT-PCR) (Taqman gene expression assays). The housekeeping gene, *Gapdh*, was used as an endogenous control. Data analysis was normalized to *Gapdh* expression, and the results were performed as relative expression divided by the basal expression level on day 0. The relative mRNA expressions of *Alpl* and *Bglap3* were shown in Fig 6(A) and 6(B). On day 4, the *Alpl* expression of the coating **5**/BMP-2 modified group reached its highest value and surpassed other groups until day 7. After day 11, the *Alpl* expressions among all testing groups were nearly the same. The *Bglap3* expression of the coating **5**/BMP-2 modified group surpassed other groups from day 4, and then achieved its maximum on day 7. *Bglap3* expressions were also no significantly different among the testing groups after day 11. The *Alpl* and *Bglap3* mRNA expressions were in accordance with the results of ALP activity and ARS staining, verifying the osteogenesis-inducing ability of coating **5**/BMP-2 modified surface. These findings suggest that the amounts of BMP-2 immobilized on surface by CVD methods should be sufficient to induce bone formation.

Bone morphogenetic protein-2 (BMP-2) is a signaling protein in the bone healing process and enhancing therapeutic efficacy [40, 41]. Therefore, coating or immobilizing BMP-2 onto surfaces is reported to enhance the osseointegration of materials [42–47]. The physically coating of BMP-2 onto surfaces has been reported in some studies [48–50]. However, since the molecules are held by weak interactions by the process, many of the molecules will diffuse from the surface without eliciting the desired response. In current clinical practice, collagen sponges have been functionalized by the adsorption of several milligrams of BMP-2 (e.g., INFUSE®) to promote the repair of large bony defects [51, 52]. However, this method is not satisfied to induce a sustained osteogenic response at the site of implantation. Because the BMP-2 release too rapidly from the surface-adsorbed depot in the collagen sponges. The difficulty cannot be overcome simply by increasing the loaded dose of BMP-2. Since the transiently high local concentration of BMP-2 could induce serious side effects, such as an over-stimulation of local bone resorption and an induction of bone formation at unintended sites [53–55]. To maximize the osteogenic efficacy and reduce the side effects, BMP-2 must be delivered to the target site gradually, at a low level and in a sustained manner, rather than in a single high-dose burst [56, 57]. Ccreating a stable covalent immobilization on the surface has been recognized as an effective way to modify orthopaedic implants. Some studies showed that there were preparations of chitosan, dextran, or polymer layers on different materials (e.g., Ti6Al4V, Stainless Steel, etc.) to covalently immobilize BMPs [58–61]. However, some methods were limited on the specific materials, such as the method of self-assembled monolayer. Continuous efforts have been devoted to the development of advanced surface coatings to realize the controlled amount of BMP-2 and to maximize their osteoinductive efficacy [62]. Several studied have immobilized BMP-2 on the surface using different methods, such as the self-assembled monolayer on gold-coated surfaces [62] or polydopamine coating [63], and the surface coverage of BMP-2 were estimated to be 70−80 ng/cm2 or 124±9 ng/cm2. The immobilization strategies have been proved to be efficient in triggering both short- and long-term osteogenic signaling responses. The experiments demonstrate that amounts as low as a nanogram of BMP-2 are effective for promoting bone formation.

Our investigations indicated that immobilized rhBMP-2 by CVD methods exhibiting the long-term effects after several days in culture, and trigger ALP expression and increase mineral production. The induction of ALP and cell mineralization clearly proves that osteogenic differentiation does not require release of the growth factor from the surface, when cells are exposed to it in a manner which allows receptor activation as described here.

## Conclusions

The immobilization of BMP-2 proteins was realized by using a novel coating of NHS ester-functionalized poly-*p*-xylylene on material surfaces, and showed sustainable and effective osteogenic activities for MC3T3-E1 preosteoblasts on such modified surfaces. The amount of immobilized BMP-2 was analyzed by QCM, and the binding affinity toward primary antibody was also examined. The coating technology based on CVD polymerization process is robust and versatile, and is equally applicable to various substrate materials. In addition, the rationale NHS ester side group is widely applicable for conjugations with proteins, enzymes, and other amine-derived biomolecules. The extension to attach these molecules is anticipated beyond the osteogenesis application demonstrated herein, and may be broadly applicable for a diverse range of materials and devices according to the specific application. The proposed one-step coating technology to install NHS ester functionality has provided a straightforward and practical approach for the design of advanced biomaterials, and is foreseeable to be applied to diagnostic devices, cellular assays, tissue engineering, and applications of regeneration medicine.
